# Editorial: Somatic comorbidities in psychiatric disorders: From childhood to old age

**DOI:** 10.3389/fpsyt.2022.1027137

**Published:** 2022-10-20

**Authors:** Eva Z. Reininghaus, Hande Sipahi, Julia Martini

**Affiliations:** ^1^Department of Psychiatry and Psychotherapeutic Medicine, Medical University of Graz, Graz, Austria; ^2^Department of Toxicology, Faculty of Pharmacy, Yeditepe University, Istanbul, Turkey; ^3^Department of Psychiatry and Psychotherapy, Faculty of Medicine, Carl Gustav Carus University Hospital, Technische Universität Dresden, Dresden, Germany

**Keywords:** somatic comorbidity, depression, diabetes, metabolic syndrome, COVID-19

Severe mental disorders such as affective disorders (including unipolar depression and bipolar disorder) and schizophrenia are psychiatric disorders with a complex multifactorial etiology. Medical comorbidities occur to a much higher extent in individuals with mental disorders compared to the general population (1) and the association between psychiatric diseases and medical comorbidities is multifaceted (2). Especially, psychopharmacological medications (3) and poor lifestyle (i.e., lack of regular physical activity, poor food intake and dietary habits, substance abuse, and high rates of smoking) (4) increase the risk of developing metabolic syndromes. Additionally, an independent association between mental disorders and physical illnesses has been proposed and linked to inflammatory pathways (5, 6). Emerging evidence also indicates that reduced cognitive functioning—and even higher risk for dementia—is associated with obesity in otherwise healthy individuals which might be an expression of abnormalities in brain structure and function (7). In recent years, studies focused especially on the possible effects of immunnomodulatory agents on mental illness for a better understanding of the underlying mechanisms of both diseases (5).

This Research Topic aims at tackling somatic comorbidity in individuals with psychiatric disorders from childhood to old age, with a focus on severe mental disorders such as anxiety, depression, bipolar disorder and schizophrenia. Neurobiological background, clinical and therapeutic aspects as well as recent developments in the content of COVID-19 infections have been included in the seven articles of this special issue.

The aim of the cross-sectional multi-center study by Yang et al. was to understand more about socioeconomic aspects and the role of somatic disorders in individuals with **anxiety and depression in general hospitals**. Married and divorced/widowed patients, patients with neurological or endocrine diseases, and those with a monthly income of ¥ 1,000–3,000 were more likely to suffer from depression. Moreover, elderly patients, patients with a senior high school or higher, and patients with a monthly income of ¥ 5,000 or above were more anxious. Patients who had experienced severe life events or cancer, as well as women were generally more often affected by a combination of anxiety and depression.

In the cross-sectional study by Pinna et al., 172 outpatients **with Type 1 diabetes** aged 17–55 years, were recruited in a special diabetes clinic to assess the relationship between depression, eating disorders, disturbed eating behavior and Type 1 diabetes. The results revealed a high prevalence of depression (35.5%), eating disorders (lifetime: 20.9%), and disturbed eating behavior (19.2%). Eating disorders were more prevalent in individuals with depression and a disturbed eating behavior increased the odds of depression significantly. This demonstrates the need for adequate screening of Type 1 diabetes in this patient population to deliver tailored treatment strategies and enhance clinical outcomes.

The work by Korczak et al. examined **risk factors for cardiovascular diseases** in a cross-sectional study of 77 children and adolescents from a pediatric depression program. About half of the participants had a family history of cardiovascular diseases and about a quarter of the participants had a BMI in overweight/obese, (pre-)hypertension or elevated cholesterol levels, respectively. Thus, cardiovascular diseases risk factor screening among children and adolescents with major depressive disorder is a valuable opportunity for prevention of future cardiovascular diseases.

The multi-center study (Germany, Austria, Denmark) by Sperling et al. examined **physical and mental health and changes in daily structure and behavior due to the COVID-19 pandemic** in individuals with bipolar disorder (*N* = 118) and healthy controls (*N* = 215). Individuals with bipolar disorder reported a statistically significant decrease in physical activity and an increased weight gain, and more physical comorbidities. Although they reported more anxiety, worries concerning health, and higher rates of COVID-19 testing, they indicated less social distancing. Overall, individuals with bipolar disorder had more difficulties compensating their behavior due to the pandemic and low-threshold professional might help to maintain daily structure and healthy dietary habit*s*.

Related to dietary habits, the high prevalence of increased BMI and rates of metabolic syndrome in individuals with psychiatric disorders are also evident. The study by Lupton-Smith et al. investigated **predictors of weight loss among individuals with serious mental illness** (*N* = 137) in an 18-month behavioral weight loss intervention. Consistent with other studies from the general population this analysis showed that early weight loss is a strong predictor of later weight loss, so early monitoring of weight is important to identify individuals who will likely lose weight or not by the end of the intervention. Therefore, it is important to track weight loss during an intervention and in clinical trials as useful indicator for overall weight loss.

Changes in weight and BMI are often associated with gastrointestinal syndromes and common individuals with mental illness. Fu et al. investigated whether gastrointestinal disorders have an effect on the fractional amplitude of low-frequency fluctuation (fALFF) patterns in patients with major depressive disorder. The study revealed that symptoms of major depressive disorder such as insomnia, anxiety/somatization, and weight loss were more prominent in patients with gastrointestinal syndrome. Moreover, increase in fALFF in the right superior frontal gyrus/middle frontal gyrus and decrease in fALFF in the left superior medial prefrontal cortex seen in these patients were considered to be one of the distinguishing markers of major depressive disorder patients with gastrointestinal syndromes.

To better understand the expression of depression and anxiety in neurological disorders, Vaccarino et al. analyzed common data elements (Demographics, self-reported symptoms of depression and anxiety) across different studies and pooled for five neurological diseases (Alzheimer's disease/amnesic mild cognitive impairment, amyotrophic lateral sclerosis, cerebrovascular disease, frontotemporal dementia, and Parkinson's disease) and major depressive disorders. By providing access to aggregated datasets on participants with and without different brain disorders, the Ontario Brain Institute's “Brain-CODE” – a large scale informatics platform – will facilitate analyses both within and across diseases and cover multiple brain disorders and a wide array of data, including clinical, neuroimaging, and molecular.

This Research Topic gathers articles carried out in different research fields to stipulate further research. The articles contain discussions regarding the current literature, recent research approaches and limitations of present studies. The discussion may help researchers and other professionals to have very different perspectives on somatic comorbidities in psychiatric disorders throughout the lifespan and to bring knowledge back to the patients.

Regarding depression and associated somatic disorder, a special focus should be laid on the prevention of cardiovascular disease in individuals with early onset of depression. Especially COVID-19 pandemic lead to lower physical activity levels and higher increase in weight in individuals with bipolar disorder compared to the general population, factors that might further increase the risk for cardiovascular disease. On the other hand, patients with neurological or endocrine disorders as well as diabetes mellitus type 2 (TD2) or lower income, were more likely to experience depression in patients in the general hospital. Developing data sharing option to compare larger populations of individuals with psychiatric disorders might help find further common pathways between somatic and psychiatric disorders over country boarders.

## Author contributions

All authors have taken part in writing the draft, editing, and reviewing the article. All authors contributed to the article and approved the submitted version.

## Conflict of interest

The authors declare that the research was conducted in the absence of any commercial or financial relationships that could be construed as a potential conflict of interest.

## Publisher's note

All claims expressed in this article are solely those of the authors and do not necessarily represent those of their affiliated organizations, or those of the publisher, the editors and the reviewers. Any product that may be evaluated in this article, or claim that may be made by its manufacturer, is not guaranteed or endorsed by the publisher.
